# Supplementary material for: Determinants of life satisfaction among women of reproductive age (15–49 years) in Bangladesh: A cross-sectional analysis
Source: PLoS One. 2022 Oct 31;17(10):e0276563. doi: 10.1371/journal.pone.0276563 (PMC9621435; doi:10.1371/journal.pone.0276563)
Supplement: S3 File — Definition of multinomial logistic regression and interpretation of relative risk ratio (RRR). (ZIP) [file pone.0276563.s003.zip › S3_File.pdf]

Nominal logistic regression

As defined by Dobson and Barnett [1], the multinomial logistic regression model is used when there is no sequential order among the levels of the dependent variable. In the multinomial logistic model, one of the K categories is chosen as the *reference category*, which is completely arbitrary. Suppose we consider the 1st category as the reference category, then the logits for the remaining $K-1$ categories can be defined as

$$\begin{aligned}\log\left(\frac{\pi_k}{\pi_1}\right) &= \delta_{0k} + \delta_{1k}x_1 + \delta_{2k}x_2 + \ldots + \delta_{pk}x_p \\ &= \mathbf{x}^T\delta, \text{ for } k = 2, \ldots, K. \end{aligned} \tag{1}$$

To find the estimates of the parameters δ, the $(K-1)$ logit equations are solved simultaneously. After obtaining the parameter estimates b_k, the linear predictors $\mathbf{x}_k^T\mathbf{b}_k$ are computed. From Equation 1, we get

$$\hat{\pi}_k = \hat{\pi}_1 \exp\left(\mathbf{x}_k^T\mathbf{b}_k\right), \text{ for } k = 2, \ldots, K.$$

Since $\hat{\pi}_1 + \hat{\pi}_2 + \ldots + \hat{\pi}_K = 1$, so

$$\hat{\pi}_1 = \frac{1}{1+\sum_{k=2}^{K}\exp\left(\mathbf{x_k^T b_k}\right)} \text{ and } \hat{\pi}_k = \frac{\exp\left(\mathbf{x}_k^T\mathbf{b}_k\right)}{1+\sum_{k=2}^{K}\exp\left(\mathbf{x}_k^T\mathbf{b}_k\right)}, \text{ for } k = 2, \ldots, K.$$

Relative risk ratio (RRR) in nominal logistic regression model

Let the outcome variable Y has three categories, that is, $y = 1, 2, 3$ and X be the explanatory variable. In the multinomial logistic regression model, $\delta^{(1)}$, $\delta^{(2)}$, $\delta^{(3)}$ are estimated, analogous to each level of the response variable:

$$\text{prob}(y=1) = \frac{\exp\left(X\delta^{(1)}\right)}{\exp\left(X\delta^{(1)}\right) + \exp\left(X\delta^{(2)}\right) + \exp\left(X\delta^{(3)}\right)},$$
$$\text{prob}(y=2) = \frac{\exp\left(X\delta^{(2)}\right)}{\exp\left(X\delta^{(1)}\right) + \exp\left(X\delta^{(2)}\right) + \exp\left(X\delta^{(3)}\right)},$$
$$\text{prob}(y=3) = \frac{\exp\left(X\delta^{(3)}\right)}{\exp\left(X\delta^{(1)}\right) + \exp\left(X\delta^{(2)}\right) + \exp\left(X\delta^{(3)}\right)}.$$

Since there are multiple solutions to $\delta^{(1)}$, $\delta^{(2)}$, and $\delta^{(3)}$, the model is unidentified, for which each outcome will have same probabilities. To solve this problem, let us arbitrarily set one of the coefficients to 0. For example, setting $\delta^{(1)} = 0$, we get

$$\text{prob}(y=1) = \frac{1}{1 + \exp\left(X\delta^{(2)}\right) + \exp\left(X\delta^{(3)}\right)},$$
$$\text{prob}(y=2) = \frac{\exp\left(X\delta^{(2)}\right)}{1 + \exp\left(X\delta^{(2)}\right) + \exp\left(X\delta^{(3)}\right)},$$
$$\text{prob}(y=3) = \frac{\exp\left(X\delta^{(3)}\right)}{1 + \exp\left(X\delta^{(2)}\right) + \exp\left(X\delta^{(3)}\right)}.$$

The relative probability of $y = 3$ to the reference category is

$$\frac{\text{prob}(y=3)}{\text{prob}(y=1)} = \exp\left(X\delta^{(3)}\right).$$

This ratio is usually called the relative risk. Let $\mathbf{X} = (x_1, x_2, \ldots, x_k)'$ and $\delta^{(\mathbf{k})} = (\delta^{(3)}, \delta^{(3)}, \ldots, \delta^{(3)})$. Then the ratio of relative risk for a single unit change in x_i is

$$\frac{\exp\left(\delta^{(3)}x_1 + \ldots + \delta^{(3)}(x_i + 1) + \ldots + \delta^{(3)}x_k\right)}{\exp\left(\delta^{(3)}x_1 + \ldots + \delta^{(3)}x_i + \ldots + \delta^{(3)}x_k\right)} = \exp\left(\delta^{(3)}\right).$$

Therefore, the exponent of δ is the relative risk ratio (RRR) for a single unit alteration in the independent variable [2]. Generally, if the RRR is greater than one, the outcome is more likely to be in the comparison group, and if the RRR is less than one, it is more likely to be in the reference group [3].

References

1. Dobson AJ, Barnett AG. An introduction to generalized linear models. Chapman and Hall/CRC; 2018.
2. StataCorp L. Stata base reference manual. College Station: StataCorp LLC. 2005.
3. Bruin J. newtest: command to compute new test @ONLINE; 2011. Available from: `https://stats.idre.ucla.edu/stata/ado/analysis/`.
